# Pathogenic germline TP53 mutations in adult sarcoma patients; implications for treatment and screening – description of an upcoming project

**DOI:** 10.1186/1897-4287-10-S2-A61

**Published:** 2012-04-12

**Authors:** K Mahendran, M Ballinger, J Kirk, D Thomas, M Tattersall

**Affiliations:** 1 The Familial Cancer Service, Westmead Hospital, NSW, Australia; 2 The University of Sydney, NSW, Australia; 3 Peter McCallum Cancer Institute, VIC, Australia; 4 Royal Prince Alfred Hospital, NSW, Australia

## 

Identification and clinical annotation of risk alleles is critical for young people with sarcoma for several reasons. The mutated gene can be passed onto the next generation affecting reproductive choices. In addition, the risk of a second malignancy appears to be markedly increased in some patients. Thirdly, the risk to other relatives, parents and siblings may vary from 50% to an unquantifiable risk. Finally, ionizing radiation increases cancer risk synergistically in the presence of mutations in sarcoma genes such as *Rb* and *TP53* and diagnostic and therapeutic radiation is modifiable in high risk individuals provided such mutations are identified.

The primary aim of this project will be seeking to establish whether the detection of pathogenic germline *TP53* mutations in adult sarcoma patients can improve outcomes through tailored clinical management. These patients have been recruited through the International Sarcoma Kindred Study (ISKS). This project will have access to the ISKS biospecimens, pedigree data, genetic information and data associated with clinical outcomes. The ISKS currently has 450 adult sarcoma probands, with recruitment on going. To date, sequencing has already been performed on 272 of these probands, in which 12 pathogenic mutations have been detected (4%).

Index cases with an identified pathogenic *TP53* mutation and their families will be systematically followed up to ascertain outcomes and to recruit further family members. The genetic status of this cohort will be collated with their clinical characteristics through examination of medical records and family history through pedigree data. The clinical outcomes of the identified pathogenic *TP53* mutation carriers and treatments given will be documented and compared against affected non-mutation carriers using a pseudo case-control study design to assess how the effects of treatment combine with mutation status.

A further aim of this project will be to conduct analyses nested within the whole ISKS cohort to seek to establish the pathogenicity of unclassified variants that have been detected so far in the ISKS cohort. This analysis will be twofold with the first part of the analysis being conducted with novel variants that have not yet been previously described. Once these variants are classified using an integrated evaluation approach, they will be examined alongside other variants detected in the cohort that have previously been described but where the magnitude of their effect on the risk of sarcoma has not yet been defined. A case-control study will then be conducted, which will be seeking to establish whether these variants are found at a higher frequency in sarcoma patients than the general population, thus implying an association with sarcoma. Based on this information, these variants will then be graded according to the guidelines recommended by the IARC Unclassified Genetic Variants Working Group to guide clinical management for the different variants.

